# Research on adults with subthreshold depression after aerobic exercise: a resting-state fMRI study based on regional homogeneity (ReHo)

**DOI:** 10.3389/fnins.2024.1231883

**Published:** 2024-03-12

**Authors:** Wenbin Shen, Xiaoxiao Wang, Qin Li, Qingguo Ding, Hongqiang Zhang, Zheng Qian, Zhixin Sun, Xingyu Chen, Jun Zhang, Mengqi Zhao, Lina Huang, Wei Xing

**Affiliations:** ^1^Department of Radiology, The Affiliated Changshu Hospital of Nantong University, Changshu No.2 People's Hospital, Changshu, Jiangsu, China; ^2^School of Foreign Studies, China University of Petroleum, Qingdao, Shandong, China; ^3^Department of Psychiatry, Changshu Third People's Hospital, Changshu, Jiangsu, China; ^4^School of Psychology, Zhejiang Normal University, Jinhua, Zhejiang, China; ^5^Department of Radiology, The Third Affiliated Hospital of Soochow University, Changzhou, Jiangsu, China

**Keywords:** subthreshold depression, aerobic exercise, neuroimaging, prevention, resting-state, regional homogeneity

## Abstract

**Objective:**

Subthreshold depression (StD)/subsyndromal depression refers to a threatening precursor to depression. Aerobic exercise is a promising self-supportive adjunctive intervention and an effective measure for StD. Our study utilizes regional homogeneity (ReHo) to investigate the impact of aerobic exercise on resting-state brain function.

**Methods:**

A total of 78 subjects, aged between 18 and 48 years, (StD group, *n* = 44; healthy control (HC) group, *n* = 34) engaged in moderate-intensity aerobic exercise 3–4 times per week for 8 weeks. Resting-state brain function and structural images were acquired before and after the exercise intervention. The ReHo method was employed to analyze abnormal changes in regional brain function, and a correlation analysis was performed using the Patient Health Questionnaire-9 (PHQ-9) and Self-Rating Anxiety Scale (SAS) scores.

**Results:**

The principal observation reveals synchronous abnormalities in the right anterior cingulate gyrus of the brain in StD subjects compared to HCs at baseline, with these differences dissipating after the implementation of aerobic exercise. After completing the aerobic exercise program, the StD group exhibited a difference in the right middle cingulate gyrus, while the left supplementary motor area (SMA) was altered in the HC group.

**Conclusion:**

Disparities in neural synchronization are evident between HCs and StD subjects, and the implementation of aerobic exercise intervention can effectively mitigate these distinctions, leading to a significant reduction in depressive symptoms among StD subjects. The primary mechanism of StD symptoms may involve the inhibition of the anterior cingulate gyrus, while the effects of aerobic exercise may be related to the modulation of neural synchronization of emotional reflexes. The discovery of these fMRI evidence findings may offer novel strategies for early detection and intervention in cases of StD.

## 1 Introduction

Depression (Cooney et al., 2013) remains a prevalent psychiatric disorder, and extensive research on depression has predominantly centered around medical interventions (American Psychiatric Association, 2010). Despite optimal treatment for all individuals, depression remains a recurring lifelong illness and the risk increases with every recurrence (Rakel, 1999). Nevertheless, primary preventive interventions play a crucial role in averting the onset of clinically significant depressive symptoms. Hence, there has been a growing interest in identifying high-risk groups and implementing timely preventive measures (Rotenstein et al., 2016).

As a threatening precursor to the major depressive disorder (MDD) (Tuithof et al., 2018), subthreshold depression (StD)/subsyndromal depression exhibits two to four criterion depressive symptoms for 2 weeks or more, including at least one core symptom (depressed mood or anhedonia) (Rodríguez et al., 2012), but does not meet the diagnostic criteria of a full-blown MDD (Pincus et al., 1999). Individuals experiencing StD face an elevated risk of progressing to a depressive disorder (Cuijpers and Smit, 2004; Hermens et al., 2004), which can lead to severe consequences for both the individual and society. However, for those experiencing StD, current evidence does not suggest that medication or psychotherapy provides significant preventive effects (Fournier et al., 2010; Cuijpers et al., 2021). Thus, there is ongoing interest in exploring alternative treatments for StD. Aerobic exercise, as one of the alternative treatments, is a promising self-supportive adjunctive intervention, and the effect of exercise on StD has been the subject of research for decades (Beesley and Mutrie, 1997). Exercise may alleviate depression through common neuromolecular mechanisms such as increased expression of neurotrophic factors [i.e., brain-derived neurotrophic factor (BDNF)], among others (Garza et al., 2004). Meta-analytic data from randomized clinical trials have demonstrated that aerobic exercise can effectively reduce depressive symptoms in StD populations (Gordon et al., 2018), with a notable emphasis on moderate-intensity aerobic exercise (Chekroud et al., 2018). Moreover, recent research indicated that aerobic exercise can prevent the onset of clinical depression symptoms (Rebar et al., 2015), although the underlying mechanisms remain unclear. Our previous analysis found that the amplitude of low-frequency fluctuation (ALFF) in the right putamen was increased in individuals experiencing STDs and that the original difference disappeared after aerobic exercise. However, our investigation did not reveal any correlation between the ALFF and the clinical scores from pre- to post-intervention (Huang et al., 2021). This suggests that the human brain functions not only through local neurons activity but also through interregional relationships (Jiang and Zuo, 2016). In a study conducted by Hwang et al. (2015), it was reported that subthreshold depression is associated with impaired resting-state functional connectivity of the cognitive control network. In our study, we aimed to assess the effect of aerobic exercise on StDs by examining the synchrony of adjacent brain regions. As a result, we speculate that ReHo may be more suitable for evaluating depression in this context.

Regional homogeneity (ReHo) is a resting-state functional magnetic resonance imaging (rs-fMRI) data-driven analysis method, which employs Kendall's coefficient of concordance (KCC) to measure the coordination of activities between voxels in certain area to reflect synchronization (Zang et al., 2004). ReHo value analysis not only reflects regions that are active but also represents coherence and centrality in some specific regions of neurons (Lv et al., 2018). Its characteristics provide an opportunity to discover localized functional disruptions (Yao et al., 2009; Wu et al., 2011). Several studies have found alterations in ReHo values within the default mode network (DMN) in depression patients, and these alterations have also been associated with a predisposition to major depressive disorder (Andreescu et al., 2013; Liston et al., 2014). Abnormal ReHo values in the cingulate gyrus and limbic regions will change with antidepressant therapy (Guo et al., 2011; Wang et al., 2014; Yang et al., 2014). To the best of our knowledge, the ReHo value analysis of the effects of aerobic exercise intervention in the StD population has not been reported yet.

In the present study, we collected brain imaging data in StD subjects and healthy controls (HCs) before and after an 8-week period of moderate-intensity aerobic exercise [which has been confirmed to be effective in decreasing depressive symptoms (Brush et al., 2020)]. Drawing upon existing literature, our hypothesis posits that coherence and centrality in the cingulate gyrus are compromised in StD patients, particularly during the processing of self-emotional stimuli, as this region is known to be most active during such emotional processing tasks (MacDonald et al., 2000; Mayberg et al., 2002). Additionally, we anticipate that the 8-week aerobic exercise program will exert a modulating effect on the brain activity within the cingulate gyrus in StD patients.

## 2 Methods

### 2.1 Subjects

The clinical data are shown in Table 1. The experiment follows a prospective, two-arm, parallel-group, controlled 2 × 2 experimental design. Specifically, all participants were informed about the longitudinal design of the study at the outset. A total of 103 volunteers (aged between 18 and 48 years) were recruited for this study (Table 1). All volunteers met the following conditions: (1) No history of receiving any psychotherapy within the past 6 months; (2) No organic disease or other serious substance addiction (such as tobacco, alcohol, or drugs); (3) No contraindications for medical maximal exercise; (4) Had engaged only in irregular exercise or low- to moderate-level habitual aerobic activity based on the Chinese version of the International Physical Activity Questionnaire-Short Form (IPAQ-SF) (Craig et al., 2003; Lee et al., 2011). We used the Patient Health Questionnaire-9 (PHQ-9) to assess the depressive symptoms of the subjects. Considering that depression often coexists with anxiety (Tiller, 2013), Self-Rating Anxiety Scale (SAS) was used to assess anxiety symptoms (Zung, 1971). These volunteers were further divided into the StD group and the HC group. The inclusion criteria for the StD group: PHQ-9 score ≥ 5 with at least one of the core symptoms (depressed mood or anhedonia) (Kroenke, 2017); The inclusion criteria for the HC group: PHQ-9 score <5. Written informed consent was obtained from all subjects. The study protocol was approved by the ethics committee of Xuzhou Medical University (2020-KY-006).

**Table 1:** Baseline demographics of all subjects.

**Variables**	**StD group**	**HC group**	***t*/*x*^2^ values**	***P* value**
	**(*****n*** = **44)**	**(*****n*** = **34)**		
Age (years)	30.66 ± 7.45	31.50 ± 10.72	0.207	0.836^a^
Sex (female/male)	20/24	13/21	0.640	0.522^b^
Education years	14.82 ± 2.18	16.23 ± 2.03	2.930	0.005^a*^
BMI (kg/m^2^)	22.16 ± 3.28	22.16 ± 3.37	0.001	0.999^a^
Marriage (married/single)	26/18	17/17	0.801	0.423^b^
IPAQ-SF, n (%)			0.367	0.714^b^
Low-level	39 (88.64%)	31 (91.18%)		
Moderate-level	5 (11.36%)	3 (8.82%)		

BMI, Body Mass Index; IPAQ-SF, International Physical Activity Questionnaire-Short Form. ^*^Represents a statistical difference.

^a^The p-value was obtained by Student's t-test; ^b^The p-value was obtained by the two-tailed Pearson chi-squared test; Low-level, no aerobic activity reported or some activity reported but not meeting the moderate-level criteria; Moderate-level, 3 or more days of vigorous activity lasting at least 20 min/day, 5 or more days of moderate- or low-intensity activity lasting at least 30 min/day, or 5 or more days of any combination of low-intensity, moderate-intensity, or vigorous activity of at least energy expenditure 600 MET·min/week.

### 2.2 Aerobic exercise

Before starting the study, we acquired rs-fMRI scans on both StD subjects and HCs, which can be referred as the baseline assessment. All subjects engaged in aerobic exercise sessions three to four times a week for a duration of 8 weeks. Each session involved moderate-intensity aerobic exercise for approximately 45 min, which included a gradual ramp-up period, followed by an adaptive 5–10 min warm-up (consisting of light aerobic exercise at 30% of heart rate reserve), and ended with a 5 min cool-down period consisting mainly of relaxation exercises. Moderate intensity was equivalent to continuous heart rate monitoring and maintaining 60%−75% of age-predicted maximal heart rate (Karvonen et al., 1957) [i.e., 220-individual's age (in years)]. This approach aligns with the recommendations of the American College of Sports Medicine (US Department of Health, 2008) and American College of Sports Medicine position stand (Garber et al., 2011). Additionally, it has been confirmed to be an effective method in decreasing depressive symptoms (Brush et al., 2020). Following the 8-week exercise intervention, another rs-fMRI scan will be performed to assess the impact of the aerobic exercise.

### 2.3 MRI data acquisition

During the rest condition session, the subjects were instructed to close their eyes, refrain from falling asleep, and avoid thinking about anything in particular. Eligible subjects underwent MRI scanning using a 3.0T GE Discovery750W MRI scanner (General Electric Medical Systems, Waukesha, WI, USA) equipped with a 16-channel head-neck united array coil (GE Healthcare). The acquisition parameters were same for all subjects. Foam cushions were used to limit the head motion, and malleable ear plugs were used to reduce noise from the scanner. The rs-fMRI was performed using T2-weighted single-shot gradient-echo planar imaging (Zoonen et al., 2014), with repetition time = 2,000 ms, echo time = 30 ms, flip angle = 90 °, field of view = 240 mm × 240 mm, matrix = 64 × 64, and slice thickness/spacing = 3.6 mm/0.2 mm, with a total of 33 slices; (2) The sagittal T1WI structure image was acquired on the Bravo sequence with repetition time = 8.464 ms, echo time = 3.248 ms, flip angle=12°, field of view = 256 mm × 256 mm, matrix = 256 × 256, and slice thickness/spacing = 1 mm/0 mm. All images were quality-controlled by a board-certified radiologist and were subsequently anonymized to protect the subjects' identities.

### 2.4 Preprocessing of rs-fMRI data and ReHo value analysis

Data preprocessing was manipulated using RESTplus v1.24 (Jia et al., 2019) (http://www.restfmri.net/forum/REST) toolkits within Matlab R2017b (The MathWorks Inc., MA). The procedure was carried out as follows: (1) The initial 10 time points were removed to avoid non-equilibrium effects of magnetization and to allow subjects to adjust to the noise of the scanner. (2) Slice timing was carried out. (3) The head motion was corrected. Subjects with excessive head motion were excluded (more than 3.0 mm of maximal translation in any direction of x, y, or z or 3.0 ° of maximal rotation) (Friston et al., 1996). (4) We used a two-step registration method, and the structural images were co-registered with the average function image after motion correction by linear transformation. New segmentation algorithm was carried out to segment the transformed structural image into the gray matter, white matter, cerebrospinal fluid, skull, extra brain and soft tissue. Then, the motion-corrected functional images were spatially normalized to Montreal Neurologic Institute (MNI) space and were resampled to 3 mm × 3 mm × 3 mm voxels. (5) Linear detrending processing and regression covariates: Linear regression analysis was utilized to further reduce other factors, such as the head motion [using the Friston24 model (Friston et al., 1996)], white matter, and CSF signal from the standardized data, that may have an impact on the research results. (6) Filtering (0.01–0.08 Hz): High-frequency signals (such as respiration and heartbeat) were removed by filtering, and the resting-state fMRI signals after low-frequency filtering reflected spontaneous neural activity, which has important physiological significance.

### 2.5 ReHo calculation

Individual ReHo maps were generated based on the Kendall's coefficient of concordance, which is computed as the correlation between the time series of each voxel and those of its nearest neighbors (Zang et al., 2004) in a voxel-wise manner. Concordance was computed on 27 voxels (including the node voxel and the 26 neighboring voxels), which is suggested as the more appropriate cluster size to cover all directions in 3D space (Jiang and Zuo, 2016). ReHo maps were then standardized by dividing individual KCC maps by their own global mean brain KCC. Finally, spatially smoothing for standardized maps was performed with a Gaussian kernel of 6-mm full-width-half-maximum (FWHM).

### 2.6 Statistical analysis

SPSS Statistics 24.0 (SPSS Inc., Chicago, IL, United States) performed two-sample *t*-tests and chi-squared tests on demographic and clinical variables. DPABI software V4.0 (Yan et al., 2016) (http://restfmri.net/forum/DPABI) was used to analyze and compare the ReHo values of the two groups of subjects. (1) A two-sample *t*-test for horizontal ReHo values differences between the StD and HC groups at baseline and follow-up. To control the confounding factors, years of education was used as a covariate. (2) A paired *t*-test was employed to assess longitudinal ReHo values differences in the StD and HC groups. Previous studies have shown that more stringent testing levels are not effective in preventing false positives (Jia et al., 2019). Regarding multiple comparison correction, we carried out Gaussian random field theory (Worsley et al., 1996; Alodat, 2011) (GRF, voxel *P* < 0.05, cluster *P* < 0.05). The ReHo value analysis comprises the results of many statistical tests, and it is necessary to correct for these multiple dependent comparisons.

## 3 Results

### 3.1 Baseline characteristics

The initial sample size was 103. However, 11 subjects were excluded from clinical interviews (1 HC subject experiencing mania, 10 StD subjects experiencing major depression); nine subjects withdrew from the project before completing the program because of personal scheduling conflicts(5 StD subjects and 4 HC subjects); two HC subjects were excluded because of excessive head motion over the course of rs-fMRI scanning at pre-intervention, 1 HC was excluded because image normalization was not possible due to different scan parameters, and 2 StD subjects were excluded because of the intracranial organic disease. This led to a total of 78 subjects (StD group, *n* = 44; HC group, *n* = 34), ranging from 18 to 48 years old (mean age 31.03 ± 8.97, 45 female patients), all of whom were right-handed. No negative effects of the interventions were observed during the trial. Detailed demographic information is shown in Table 1.

StD subjects and HCs underwent a baseline rs-fMRI scan. There were no significant differences between the two groups with respect to age, sex, educational attainment, or body mass index (BMI). Subjects were also balanced at screening for active vs. non-active aerobic activity using the IPAQ-SF at baseline (all *P* > 0.05).

### 3.2 Resting-state fMRI data

#### 3.2.1 Regional homogeneity differences between groups before aerobic exercise (baseline)

**Figure 1:**
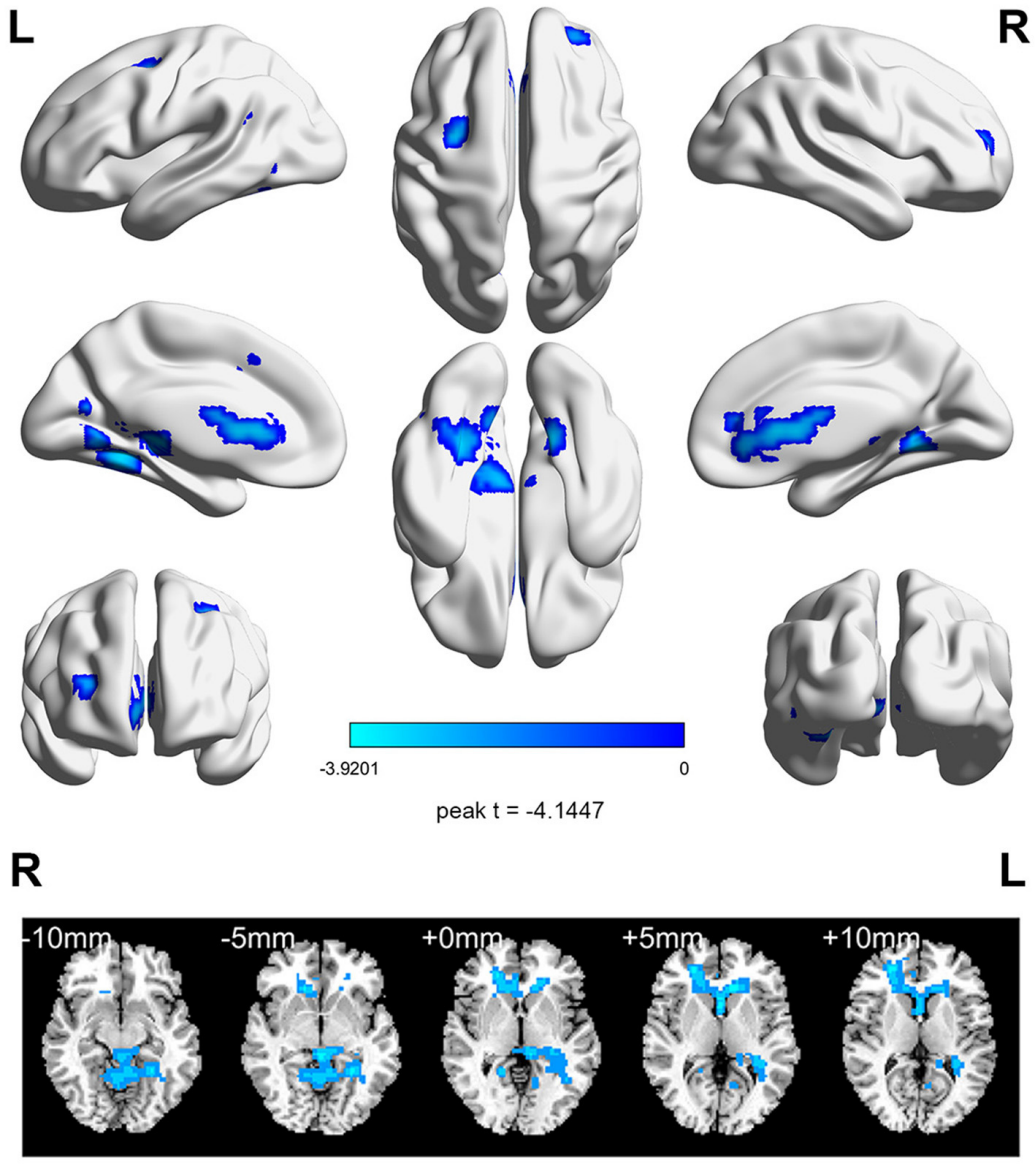
The horizontal ReHo value differences between the StD and HC groups at baseline. Cool (blue) colors represent decreased *t*-value.

Before aerobic exercise, an initial group analysis was conducted on ReHo values to explore the differences in regional activity between the StD group and the HC group. The results indicated that StD subjects showed decreased ReHo values in the right anterior cingulate gyrus compared to HCs. The results are shown in Table 2, Figure 1.

**Table 2:** Regions showing ReHo differences between the StD and HC groups at baseline.

	**Peak Location(AAL)**	**BA**	**Number of voxels**	**Peak *t* value**	**MNI coordinates**		
					**X**	**Y**	**Z**
Cluster1	Cingulum_Ant_R	25	935	−4.1447	−12	27	3

AAL, Anatomical Automatic Labeling; BA, Brodmann area; MNI, Montreal Neurological Institute; Cingulum_Ant_R, right anterior cingulate gyrus. Clusters located in the white matter were not reported.

#### 3.2.2 Regional homogeneity differences between groups after aerobic exercise

After the aerobic exercise intervention, no significant difference in ReHo values was observed between the StD and HC groups after applying GRF correction.

#### 3.2.3 Regional homogeneity differences in HCs from pre- to post- aerobic exercise intervention

**Figure 2:**
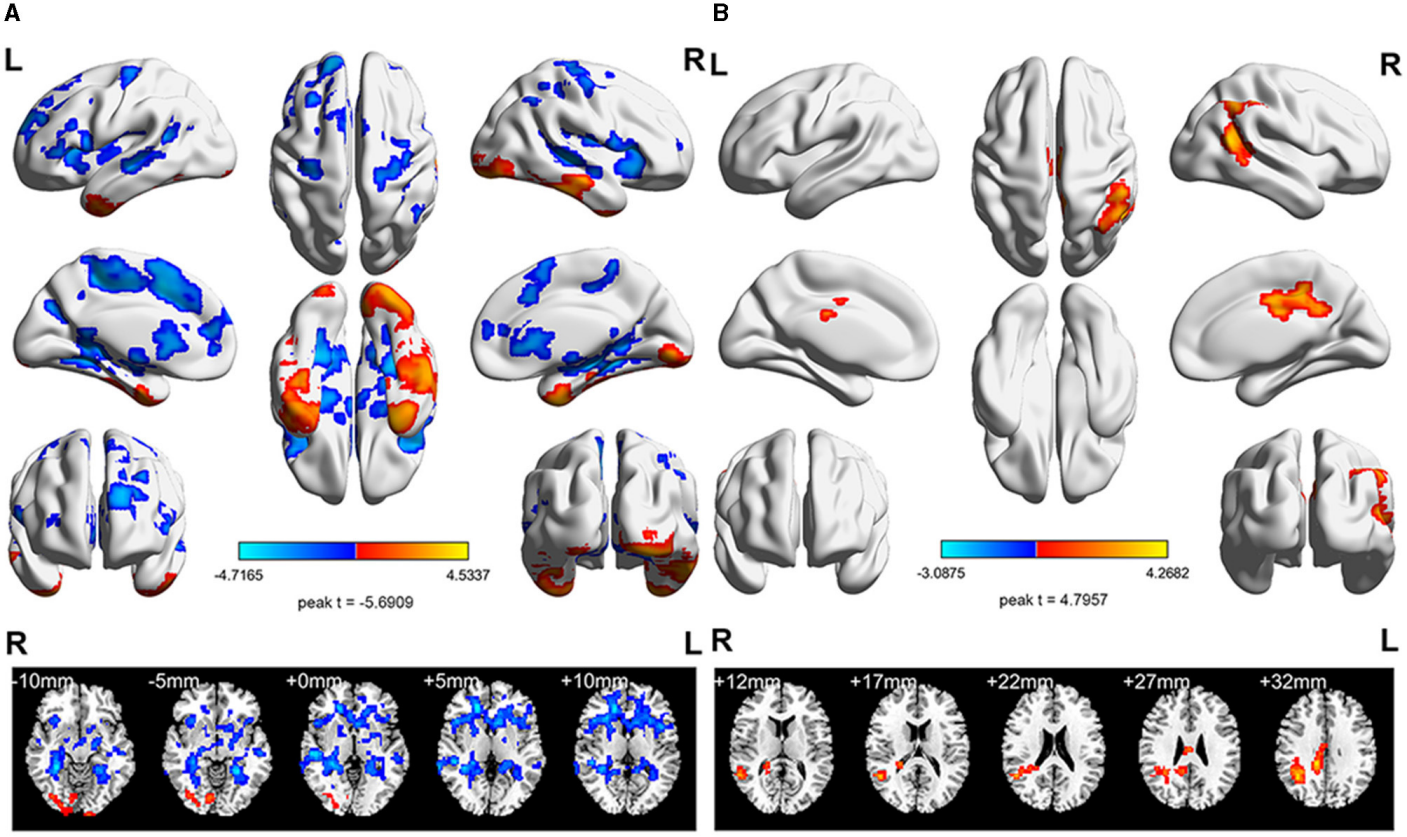
The longitudinal ReHo value differences in the StD and HC groups. **(A)** Intra-group analyses of the ReHo value in the HC group. Compared with the baseline period, the ReHo value in the left SMA decreased after aerobic exercise intervention. **(B)** Intra-group analyses of the ReHo value in the StD group. Compared with the baseline period, the ReHo value in the right middle cingulate gyrus increased after aerobic exercise intervention. Cool (blue) colors represent decreased *t*-value, whereas warm (red) colors represent increased *t*-value.

The ReHo differences of HCs before aerobic exercise and after aerobic exercise were compared. The results showed significantly decreased ReHo values in the left supplementary motor area (SMA) (Table 3, Figure 2).

**Table 3:** Regions showing ReHo value differences in the StD and HC groups from pre- to post-aerobic exercise intervention.

	**Peak Location (AAL)**	**BA**	**Number of voxels**	**Peak *t* value**	**MNI coordinates**		
					**X**	**Y**	**Z**
	**HCs from pre- to post- aerobic exercise intervention**						
Cluster1	Supplementary Motor Area_L	6	8460	−5.6909	18	36	6
	**StDs from pre- to post- aerobic exercise intervention**						
Cluster1	Cingulum_Mid_R	N/A	855	4.7957	12	−33	36

AAL, Anatomical Automatic Labeling; BA, Brodmann area; MNI, Montreal Neurological Institute; Supplementary Motor Area_L, left supplementary motor area; Cingulum_Mid_R, right middle cingulate gyrus.

Clusters located in the white matter were not reported.

#### 3.2.4 Regional homogeneity differences in the std group from pre- to post- aerobic exercise intervention

Comparing the difference in ReHo values of the StD group before and after aerobic exercise, the results showed an increased ReHo value in the right middle cingulate gyrus after aerobic exercise (Table 3, Figure 2).

### 3.3 The effect of aerobic exercise on clinical depression symptom changes

After the aerobic exercise intervention, the PHQ-9 and SAS scores were significantly improved in the StD group; the SAS scores were significantly improved; however, there was no obvious change in the PHQ-9 scores in the HC group (Table 4).

**Table 4:** Comparison of PHQ-9 and SAS scores before and after the aerobic exercise intervention.

**Groups**	**PHQ-9**		***P* value**	**SAS**		***P* value**
	**Pre-**	**Post-**		**Pre-**	**Post-**	
StD	9.37 ± 2.75	4.92 ± 3.40	<0.001^*^	46.13 ± 7.45	37.13 ± 6.29	<0.001^*^
HC	2.97 ± 2.35	2.31 ± 2.04	0.237	37.13 ± 5.89	33.16 ± 5.39	0.007^*^

PHQ-9, Patient Health Questionnaire-9; SAS, Self-Rating Anxiety Scale; ^*^represents a statistical difference.

### 3.4 Correlations between clinical scale scores and the ReHo values of brain regions

We analyzed the right anterior cingulate gyrus, which showed ReHo value differences between the StD and HC groups at baseline. However, no differences were found either (P > 0.05) (Table 5).

**Table 5:** Correlations between clinical scale scores and the ReHo values of brain regions at baseline.

**Brain Areas**	**PHQ-9**		**SAS**	
	***r***	***P* value**	***r***	***P* value**
Cingulum_Ant_R	0.018	0.909	0.012	0.941

PHQ-9, Patient Health Questionnaire-9; SAS, Self-Rating Anxiety Scale; Cingulum_Ant_R, right anterior cingulate gyrus.

Although significant changes in ReHo values have been found in the right middle cingulate gyrus in the StD group from pre- to post- exercise, ReHo values were not associated with depression or anxiety scale scores (*P* > 0.05) (Table 6).

**Table 6:** Correlations between clinical scale scores and the ReHo values of brain regions within the StD group after aerobic exercise.

**Brain Areas**	**PHQ-9**		**SAS**	
	***r***	***P* value**	***r***	***P* value**
Cingulum_Mid_R	−0.122	0.428	−0.057	0.712

PHQ-9, Patient Health Questionnaire-9; SAS, Self-Rating Anxiety Scale; Cingulum_Mid_R, right middle cingulate gyrus.

## 4 Discussion

The primary objective of this study was to explore the relationship between aerobic exercise and depressive symptoms by comparing ReHo values. To the best of our knowledge, this is the first whole-brain voxel-wise analysis conducted to evaluate regional homogeneity in StD subjects compared with HCs. The primary finding indicates synchronous abnormalities in the right anterior cingulate gyrus of the brain among StD patients compared to HCs at baseline, which subsequently diminish after the implementation of aerobic exercise. Additionally, the StD group after completing the aerobic exercise program showed a difference in the reward response area. Exploratory research suggested that aerobic exercise reduced the scores on depression and anxiety scales. Therefore, we posit that aerobic exercise does influence neural synchronization, leading to the alleviation of depressive and anxiety symptoms.

At baseline, we observed a significantly decreased ReHo values in the right anterior cingulate gyrus in the StD group compared to HCs. The anterior cingulate gyrus is widely acknowledged to play a role in regulating social reward or punishment processes (Martins et al., 2021). When its function is inhibited, the reduced responsiveness of the social reward system leads to less pleasure from social interactions. According to Yun et al., subdued reward processes may be biological markers for the increased risk of depression, prolonged suppression may further promote the progression to depression (Yun et al., 2022). Structural studies have primarily shown that depression is associated with reduced volumes, thickness, and surface area in the anterior cingulate gyrus (McLaren et al., 2016). Therefore, the baseline results, showing lower ReHo values in the right anterior cingulate gyrus and indicating impaired local-regional coherence, hold potential significance in the identification of StD.

After 8 weeks of aerobic exercise, no significant differences in ReHo values were observed in brain regions between the StD and HC groups. The results suggested that the regularity of aerobic exercise intervention contributed to the convergence of differences between the two groups. We further studied the changes in brain activity from pre- to post-aerobic exercise intervention. We found that the changes in the spontaneous brain regional activity were involved two functional areas of the brain (the right middle cingulate gyrus and the left SMA). The right middle cingulate gyrus exhibited increased ReHo values in the StD group after aerobic exercise. In general, the middle cingulate gyrus is involved in emotional reflection and regulation (Etkin and Schatzberg, 2011). Specifically, its increased activity was reflected in altered attention, observation, and response to rewards in individuals experiencing StD after aerobic exercise (Allman et al., 2001). This suggests that aerobic exercise indeed induces changes in certain functionally inhibitory regions among individuals experiencing StD.

To further explore its relationship with clinical symptoms, we conducted a correlation study between ReHo and clinical scores before and after the intervention. However, we did not find any correlation between ReHo values and clinical scores from pre- to post-intervention, maybe due to the small number of subjects. However, these results enrich our understanding of the neural basis of anhedonia in StD patients. Furthermore, our study showed decreased ReHo values in the left SMA after aerobic exercise in the HCs, indicating that aerobic exercise also has a positive effect on brain plasticity in the HCs. The SMA belongs to the sensorimotor network and is associated with sensorimotor processing and attention (Passingham et al., 2010; Peterson and Ferris, 2019). Previous research conducted by Mehren reported that moderate aerobic exercise could improve functionality of the SMA in the go/no-go task (Mehren et al., 2019). Sijie Yi discovered that reduced ReHo values in the left SMA are positively associated with reduced depressive symptoms (Yi et al., 2022), indicating that aerobic exercise is an effective neuromodulation method for reduced depressive symptoms.

The ReHo value changes in the above-mentioned brain regions during the pre- and post-aerobic exercise interventions reported by our research may indicate that aerobic exercise can adjust the circuits related to attention and response to reward. Despite its potential implications, there were still several limitations that should be acknowledged in this study. Initially, the assessment of StDs in the present study was primarily based on the PHQ-9 scores (Kang et al., 2020), a face-to-face interview, and a self-report questionnaire. Although this structured clinical interview is widely used, it may have introduced some social desirability bias and may have impacted the findings. Subsequently, the exercise trial was short-term and the aerobic exercise forms are different. Ultimately, the final sample size was relatively small (n = 44 in the StDs) because this study was a longitudinal trial, and subjects were lost during aerobic exercise intervention. While similar to our ALFF study, ReHo analysis also showed that the difference disappeared after exercise; however, due to the small sample size, we cannot exclude that the difference may not actually disappear but is simply not reflected in the ReHo indicator. Our subsequent research will delve into the discovered reward circuit, investigating the extent to which physical exercise interventions can relieve or even help StD patients in recovering from depressive symptoms (e.g., intervention duration and type). A large sample size may provide more reliable conclusions. Furthermore, we only included adults aged between 18 and 48 years as our subjects, and most of them were women. This may imply that the effects of age and sex were not completely ruled out.

In summary, our findings indicate that aerobic exercise modulates brain synchronization activity among individuals experiencing StD. Specifically, the initial differences observed in the right anterior cingulate gyrus disappeared, while a regional neural synchronization difference emerged in the right middle cingulate gyrus. The inhibition of the anterior cingulate gyrus emerges as a potential primary mechanism underlying StD symptoms, potentially leading to abnormalities in social reward or punishment processes. The efficacy mechanism of aerobic exercise may be related to the modulation of the neural synchronization of emotional reflexes. However, these insights warrant further confirmation through additional studies. Regional neural synchrony emerges as a promising novel strategy for StD detection and early intervention markers.

## Data availability statement

The raw data supporting the conclusions of this article will be made available by the authors, without undue reservation.

## Ethics statement

The studies involving humans were approved by Ethics Committee of Xuzhou Medical University (2020-KY-006). The studies were conducted in accordance with the local legislation and institutional requirements. Written informed consent for participation was not required from the participants or the participants' legal guardians/next of kin in accordance with the national legislation and institutional requirements.

## Author contributions

WS, XW, WX, and LH conceived and designed the experiments and contributed to the writing of the manuscript. HZ, ZQ, ZS, and JZ performed the experiments and analyzed the data. All authors contributed to the article and approved the submitted version.
