# Supplementary material for: Research on adults with subthreshold depression after aerobic exercise: a resting-state fMRI study based on regional homogeneity (ReHo)
Source: Front Neurosci. 2024 Mar 12;18:1231883. doi: 10.3389/fnins.2024.1231883 (PMC10963409; doi:10.3389/fnins.2024.1231883)
Supplement: Supplementary file 1 [file Data_Sheet_1.pdf]

Supplementary Material

Supplementary Tables and Figures

Table S1: Regions showing ReHo differences between the StD and HC groups at baseline (GRF correction with voxel $P < 0.01$, cluster $P < 0.05$)

	Peak Location (AAL)	BA	Number of voxels	Peak *t* value	MNI coordinates		
					X	Y	Z
Cluster1	Frontal_Mid_R	N/A	302	4.1447	-12	27	3

AAL, Anatomical Automatic Labeling; BA, Brodmann Aarea; MNI, Montreal Neurological Institute; Frontal_Mid_R, middle frontal gyrus.

Table S2: Regions showing ReHo value differences in the StD and HC groups from pre- to post-aerobic exercise intervention (GRF correction with voxel $P < 0.01$, cluster $P < 0.05$)

	Peak Location (AAL)	BA	Number of voxels	Peak *t* value	MNI coordinates		
					X	Y	Z
	**HCs from pre- to post- aerobic exercise intervention**						
Cluster1	Fusiform_R	N/A	502	-5.0927	33	-42	9
Cluster2	Insula_R	N/A	1108	-5.6909	18	36	6
Cluster3	Rolandic_Oper_L	48	1059	-5.2384	-42	-3	15
	**StDs from pre- to post- aerobic exercise intervention**						
Cluster1	SupraMarginal_R	40	192	4.2112	48	-39	42

AAL, Anatomical Automatic Labeling; BA, Brodmann Aarea; MNI, Montreal Neurological Institute; Fusiform_R, right fusiform gyrus; Insula_R, right insula; Rolandic_Oper_L, left rolandic operculum; SupraMarginal_R, right supramarginal gyrus.

Table S3: Regions showing ReHo value differences in the StD and HC groups from pre- to post-aerobic exercise intervention (GRF correction with voxel $P < 0.005$, cluster $P < 0.05$)

	Peak Location (AAL)	BA	Number of voxels	Peak t value	MNI coordinates		
					X	Y	Z
	**HCs from pre- to post- aerobic exercise intervention**						
Cluster1	Fusiform_R	N/A	342	-5.0927	33	-42	9
Cluster2	Supp_Motor_Area_L	32	589	-4.5745	-15	21	33
Cluster3	Insula_R	N/A	620	-5.6909	18	36	6
	**StDs from pre- to post- aerobic exercise intervention**						
Cluster1	SupraMarginal_R	40	133	4.2112	48	-39	42

AAL, Anatomical Automatic Labeling; BA, Brodmann Aarea; MNI, Montreal Neurological Institute; Fusiform_R, right fusiform gyrus; Supp_Motor_Area, left supplementary motor area; Insula_R, right insula; SupraMarginal_R, right supramarginal gyrus.

Table S4: Regions showing ReHo value differences in the StD and HC groups from pre- to post-aerobic exercise intervention (GRF correction with voxel $P < 0.001$, cluster $P < 0.05$)

	Peak Location (AAL)	BA	Number of voxels	Peak t value	MNI coordinates		
					X	Y	Z
	**HCs from pre- to post- aerobic exercise intervention**						
Cluster1	Fusiform_R	N/A	209	4.6444	24	-6	-42
Cluster2	Caudate_R	48	138	-5.5524	24	21	30
	**StDs from pre- to post- aerobic exercise intervention**						
			N/A				

AAL, Anatomical Automatic Labeling; BA, Brodmann Aarea; MNI, Montreal Neurological Institute; Fusiform_R, right fusiform gyrus; Caudate_R, right caudate nucleus.

Table S5: Correlations between clinical scale scores and the FC values of brain regions showing significant group differences at baseline

Brain Areas	PHQ-9		SAS	
	r	***P*-value**	*r*	***P*-value**
Cingulum_Ant_R	0.018	0.909	0.012	0.941

Cingulum_Ant_R, right anterior cingulate and paracingulate gyri; PHQ-9, the Patient Health Questionnaire-9; SAS, Self-Rating Anxiety Scale.

Table S6: Correlations between clinical scale scores and the FC values of brain regions showing significant differences within StD group after aerobic exercise

Brain Areas	PHQ-9		SAS	
	r	***P*-value**	*r*	***P*-value**
Cingulum_Mid_R	-0.122	0.428	-0.057	0.712

Cingulum_Mid_R, right middle cingulate gyrus; PHQ-9, the Patient Health Questionnaire-9; SAS, Self-Rating Anxiety Scale.

Table S7: Multiple brain regions for GRF correction with voxel $P < 0.05$, cluster $P < 0.05$

	Peak Location (AAL)	Number of voxels	Peak t value	MNI coordinates		
				X	Y	Z
	**Group differences before exercise intervention**					
Cluster1	Cingulum_Ant_R	935	-4.1447	-12	27	3
	Caudate_R	28				
	Frontal_Mid_R	24				
	Caudate_L	20				
	Frontal_Mid_L	18				
	Frontal_Sup_R	15				
	**HCs from pre- to post- aerobic exercise intervention**					
Cluster1	Supplementary Motor Area_L	8460	-5.6909	18	36	6
	Frontal_Sup_L	209				
	Frontal_Mid_L	173				

	Caudate_L	170				
	Insula_R	165				
	Precentral_R	165				
	Insula_L	161				
	**StDs from pre- to post- aerobic exercise intervention**					
Cluster1	Cingulum_Mid_R	855	4.7957	12	-33	36
	Angular_R	121				
	Temporal_Mid_R	96				
	Parietal_Inf_R	76				
	Cingulum_Post_R	25				

AAL, Anatomical Automatic Labeling; BA, Brodmann Aarea; MNI, Montreal Neurological Institute; Cingulum_Ant_R, right anterior cingulate gyrus; Caudate_R, right caudate nucleus Frontal_Mid_R, right middle frontal gyrus; Caudate_L, left caudate nucleus; Frontal_Mid_L, left middle frontal gyrus; Frontal_Sup_R, right superior frontal gyrus, dorsolateral; Supplementary Motor Area_L, left supplementary motor area; Frontal_Sup_L, left superior frontal gyrus, dorsolateral; Frontal_Mid_L, left Middle frontal gyrus; Caudate_L, left caudate nucleus; Insula_R, right insula; Precentral_R, right precentral gyrus; Insula_L, left insula; Cingulum_Mid_R, right middle cingulate gyrus; Angular_R, right angular gyrus; Temporal_Mid_R, right middle temporal gyrus; Parietal_Inf_R, right inferior parietal gyrus; Cingulum_Post_R, right posterior cingulate gyrus.

Figure S1: Brain regions showing significant differences under various correction thresholds

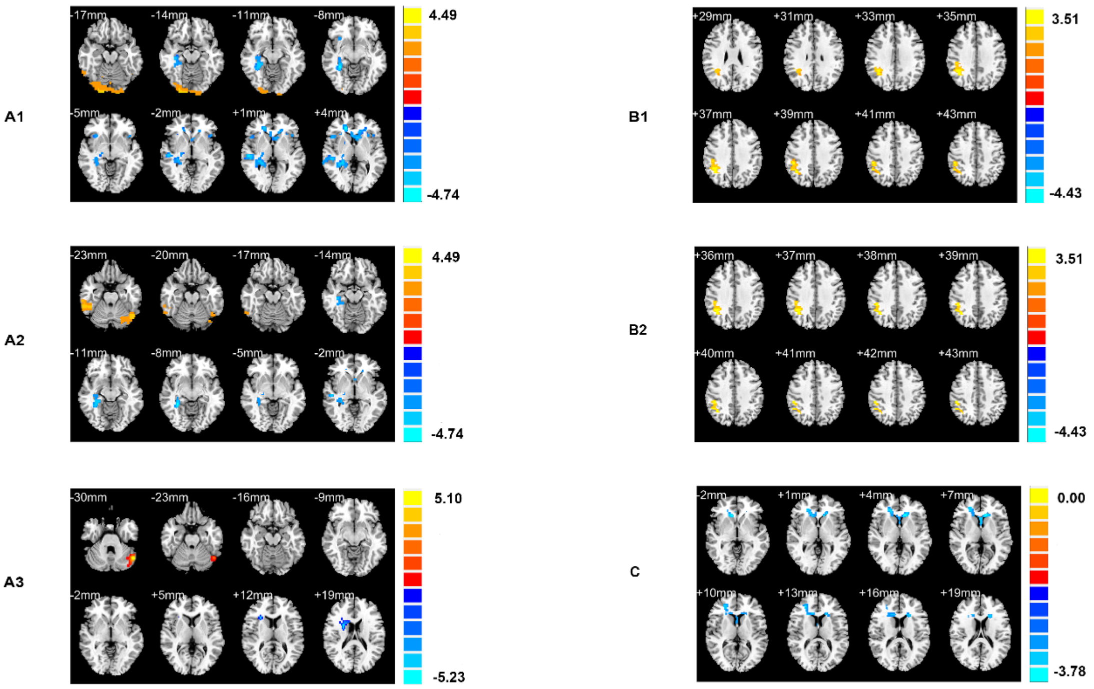

A1, HCs pre- to post- aerobic exercise intervention in voxel $P < 0.01$; A2, HCs pre- to post- aerobic exercise intervention in voxel $P < 0.005$; A3, HCs pre- to post- aerobic exercise intervention in voxel $P < 0.001$; B1, StDs pre- to post- aerobic exercise intervention in voxel $P < 0.01$; B2, StDs pre- to post- aerobic exercise intervention in voxel $P < 0.005$; C, StD and HC groups at baseline in voxel $P < 0.01$
